# Higher polymerase activity of a human influenza virus enhances activation of the hemagglutinin-induced Raf/MEK/ERK signal cascade

**DOI:** 10.1186/1743-422X-4-134

**Published:** 2007-12-05

**Authors:** Henju Marjuki, Hui-Ling Yen, John Franks, Robert G Webster, Stephan Pleschka, Erich Hoffmann

**Affiliations:** 1 Division of Virology, Department of Infectious Diseases, St. Jude Children's Research Hospital, Memphis, TN 38105, USA; 2 Department of Pathology, University of Tennessee, Memphis, TN 38105, USA; 3 Institute for Medical Virology, Justus-Liebig University, Gießen 35392, Germany

## Abstract

Influenza viruses replicate within the nucleus of infected cells. Viral genomic RNA, three polymerase subunits (PB2, PB1, and PA), and the nucleoprotein (NP) form ribonucleoprotein complexes (RNPs) that are exported from the nucleus late during the infectious cycle. The virus-induced Raf/MEK/ERK (MAPK) signal cascade is crucial for efficient virus replication. Blockade of this pathway retards RNP export and reduces virus titers. Hemagglutinin (HA) accumulation and its tight association with lipid rafts activate ERK and enhance localization of cytoplasmic RNPs. We studied the induction of MAPK signal cascade by two seasonal human influenza A viruses A/HK/218449/06 (H3N2) and A/HK/218847/06 (H1N1) that differed substantially in their replication efficiency in tissue culture. Infection with H3N2 virus, which replicates efficiently, resulted in higher HA expression and its accumulation on the cell membrane, leading to substantially increased activation of MAPK signaling compared to that caused by H1N1 subtype. More H3N2-HAs were expressed and accumulated on the cell membrane than did H1N1-HAs. Viral polymerase genes, particularly H3N2-PB1 and H3N2-PB2, were observed to contribute to increased viral polymerase activity. Applying plasmid-based reverse genetics to analyze the role of PB1 protein in activating HA-induced MAPK cascade showed that recombinant H1N1 virus possessing the H3N2-PB1 (rgH1N1/H3N2-PB1) induced greater ERK activation, resulting in increased nuclear export of the viral genome and higr virus titers. We conclude that enhanced viral polymerase activity promotes the replication and transcription of viral RNA leading to increased accumulation of HA on the cell surface and thereby resulting in an upregulation of the MAPK cascade and more efficient nuclear RNP-export as well as virus production.

## Background

Influenza viruses are members of the Orthomyxoviridae family of RNA viruses and are grouped into types A, B, and C on the basis of their nucleoprotein (NP) and matrix protein characteristics. Type A influenza viruses (IVAs) are classified into subtypes based on two proteins on the surface of the virus, hemagglutinin (HA) and neuraminidase (NA). IVAs infect a large variety of mammals and birds, occasionally producing devastating pandemics in humans [1]. Epidemics frequently occur between pandemics as a result of gradual antigenic change in the prevalent virus; this phenomenon is termed antigenic drift [2]. Currently, human influenza epidemics are caused by H1N1 and H3N2 IVAs or by type B influenza viruses (IVBs) [1,3]. Three notable (1918, 1958 and 1968) severe pandemics have occurred during the 20^th ^century: An H1N1 IVA caused the 1918 "Spanish flu" pandemic, while an H3N2 IVA was responsible for the 1968 "Hong Kong flu" pandemic [4,5]. Since the appearance of H3N2 in 1968 and the reappearance of H1N1 in 1977, IVAs have continued to circulate in humans. Although infection with either of these strains appears to have similar clinical manifestations in humans and other mammals (e.g., swine), many reports suggest that influenza caused by H3N2 viruses is usually more severe than that caused by H1N1 subtype [6].

The IVA genomes consist of eight single-stranded RNA segments of negative polarity that encode up to 11 proteins [7,8]. These RNA segments are associated with the NP and the RNA-dependent RNA polymerase, which comprises three polymerase subunits (PB1, PB2, and PA) to form viral ribonucleoprotein complexes (RNPs), representing the minimal set of infectious viral structures. Influenza viruses pursue a nuclear-replication strategy; thus, the RNPs must be exported from the nucleus to the cytoplasm to be enveloped with other viral proteins at the cell membrane [7,8].

The cellular response to growth factors, inflammatory cytokines, and other mitogens is often mediated by receptors that are either G protein-linked or intrinsic protein tyrosine kinases [9]. The binding of ligand to receptor transmits a signal to one or more cascades of serine/threonine kinases that utilize sequential phosphorylation to transmit and amplify the signal [10-13]. These kinase cascades are collectively known as mitogen-activated protein kinase (MAPK) signaling cascades [11,14]. The Raf/MEK/ERK pathway represents one of the best-characterized MAPK signaling pathways. MAPK cascades are key regulators of cellular responses such as proliferation, differentiation, and apoptosis [15]. Many negative-strand RNA viruses induce cellular signaling through MAPK cascades [16-18]. Infection with IVAs or IVBs upregulates the Raf/MEK/ERK cascade to support virus replication within the infected host cells [19-22]. This signal cascade, which is activated late during influenza infection, is essential for efficient export of nuclear RNPs. MEK inhibition has been shown to impair the nuclear RNP export and reduces virus yields [23].

Recently, we demonstrated that HA accumulation at the cell membrane and its tight association with lipid-raft domains trigger virus-induced ERK activation [24], showing an important role of HA as a viral inducer of MAPK signaling. Although HA appears to be important, we cannot exclude the involvement of other viral proteins or processes in activating MAPK signaling. In this study, we examined the activation levels of MAPK signaling induced by two currently circulating human strains: A/Hong Kong/218847/06 (H1N1) and A/Hong Kong/218449/06 (H3N2). These viruses were isolated from two different patients in Hong Kong in 2006. We observed that the H3N2 strain replicates more efficiently in tissue culture than does the H1N1 and also induced higher levels of ERK phosphorylation. The purpose of this study was to investigate whether higher viral replication efficiency is functionally connected to stronger virus-induced MAPK activation leading to enhanced nuclear RNP export and to analyze the possible contribution of viral polymerase proteins to HA-induced ERK activation.

## Results

### Human influenza virus A/HK/218449/06 (H3N2) replicates faster than A/HK/218847/06 (H1N1)

**Figure 1:**
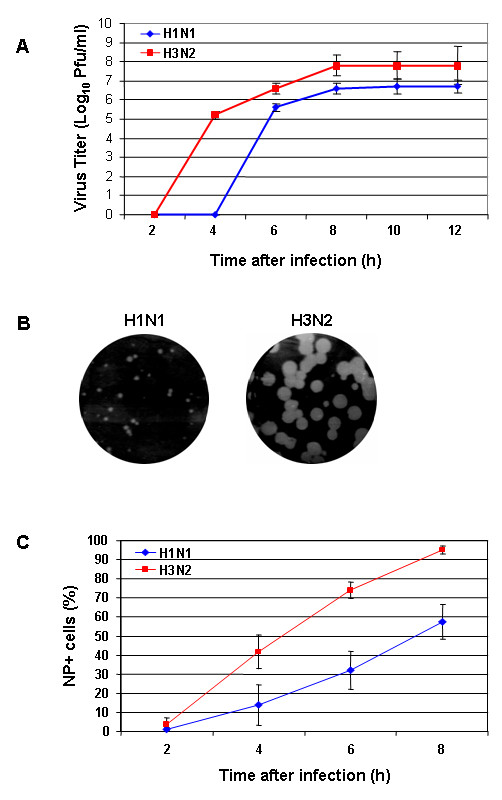
**Growth properties of A/HK/218847/06 (H1N1) and A/HK/218449/06 (H3N2) influenza viruses**. (A) MDCK cells were infected with either H1N1 virus (blue line) or H2N3 virus (red line) at m.o.i. = 2. The growth curve is based on virus titers at the indicated time points after infection. The mean virus titers are given as log_10 _plaque forming units/ml. The error bars were derived from three independent experiments. (B) Plaque formation after virus titration on MDCK cells. The virus-containing supernatant from cells infected at m.o.i. = 1 was harvested 9 h after infection. (C) MDCK cells were infected with either virus at m.o.i. = 1. The percentage of NP-expressing cells was measured by flow cytometry (FACS) using anti-NP mAb. The error bars were derived from three independent experiments.

We characterized H1N1 and H3N2 IVAs isolated from two patients in Hong Kong in 2006. MDCK cells were infected with either virus to determine the TCID_50_, viral growth, and the level of viral protein synthesized during infection. Logarithmic differences of viral infectivity titers were determined 3 days after infection via serial dilution. Infection with the H3N2 virus resulted in 2 log higher TCID_50_/ml than that seen with the H1N1 infection, which indicated higher production of infectious progeny virions of the H3N2 subtype. To determine the viral growth curve, we infected MDCK cells with either virus at m.o.i. = 2. New infectious progeny virions of H3N2 IVA were released within 4 h after infection, whereas almost no H1N1 virus could be detected within this time frame. Furthermore, a clear, at least 1 log increase in virus titers was observed in H3N2-infected cells between 6 to 12 h post infection (p.i.) (Fig. 1A). Additionally, a standard plaque assay was used to analyze plaque morphology of MDCK cells infected at m.o.i. = 1 after 3 days of incubation. The H3N2 virus formed predominantly larger plaques (diameter, 2.85 ± 0.71 mm) than that produced by the H1N1 (diameter, 1.22 ± 0.53 mm) (Fig. 1B) showing that the H3N2 subtype possesses the capability to spread faster.

To evaluate whether the amount of viral proteins synthesized during infection differed between these two strains, we measured NP production at different times in MDCK cells infected at m.o.i. = 1. Flow cytometry analysis revealed that the H3N2 IVA produced markedly more NP than did the H1N1 at 4, 6, and 8 h p.i. (Fig. 1C). Whole-cell populations infected with H1N1 showed 14% of the cells were NP-expressing; at 4 h p.i., whereas 42% of the whole-cell populations in the H3N2-infected cells were NP^+^. Around 40% more viral NP was found in H3N2-infected cells at 6 h p.i. and almost all of the cells were infected by H3N2 at 8 h p.i. This finding showed optimal replication of newly formed progeny virions of the H3N2 subtype. The amount of NP^+ ^cells at 8 h after H1N1 infection was lower than that at 6 h after infection with H3N2. Overall, our results clearly showed that the studied H3N2 virus possesses better growth capacity and replicates more efficiently in tissue culture model than does the H1N1 subtype.

### Infection with A/HK/218449/06 (H3N2) influenza virus induces stronger ERK phosphorylation and increased nuclear RNP export

**Figure 2:**
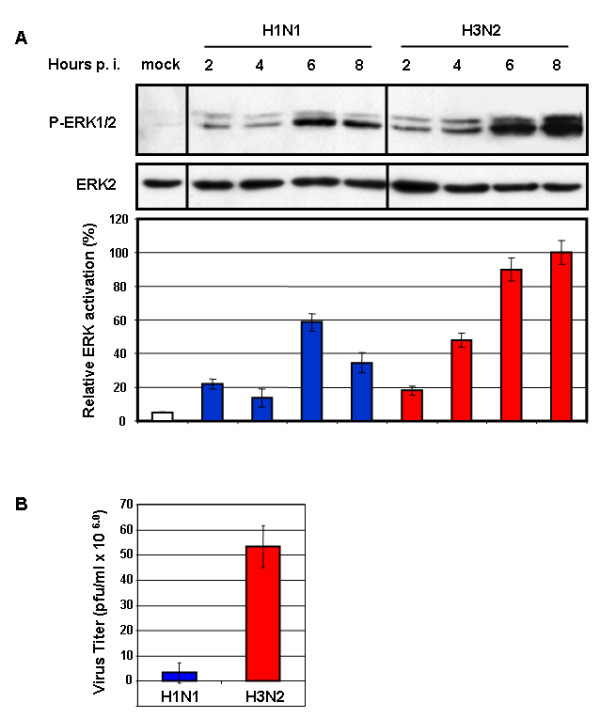
**A/HK/218449/06 (H3N2) influenza virus induces greater ERK phosphorylation leading to higher virus titers**. (A) MDCK cells were infected with either virus at m.o.i. = 1. After Western blot analysis, ERK activation was analyzed with a mAb specific for the phosphorylated kinase (P-ERK). Subsequently, loading was controlled with a mAb against ERK2. Respective bands of three independent experiments were quantified, and relative ERK activation was calculated and normalized to the loading control (mock-infected, white bar). Virus types and the time of analysis post infection (p.i.) are indicated. (B) MDCK cells were infected with either virus at m.o.i. = 1, and the supernatant was harvested at 9 h p.i. to determine the virus titers. The mean virus titers are given as plaque forming units/ml. The error bars were derived from three independent experiments.

Induction of MAPK signaling is essential for influenza virus RNP export [23]. As the H3N2 and H1N1 viruses differed substantially in their replication efficiency in tissue culture, we further examine the levels of MAPK induction and concomitantly nuclear RNP export. MDCK cells infected (m.o.i. = 1) with either type of virus were analyzed for ERK phosphorylation (activation) at different time points p.i.. The virus-induced ERK activation found in H3N2-infected cells was significantly stronger than that in H1N1-infected cells at late time points after infection (6 h and 8 h p.i.) (Fig. 2A). A reduction of H1N1-induced ERK activation was observed at 8 h p.i., a time point when ERK activation usually increases, as seen in cells infected with H3N2 (Fig. 2A).

**Figure 3:**
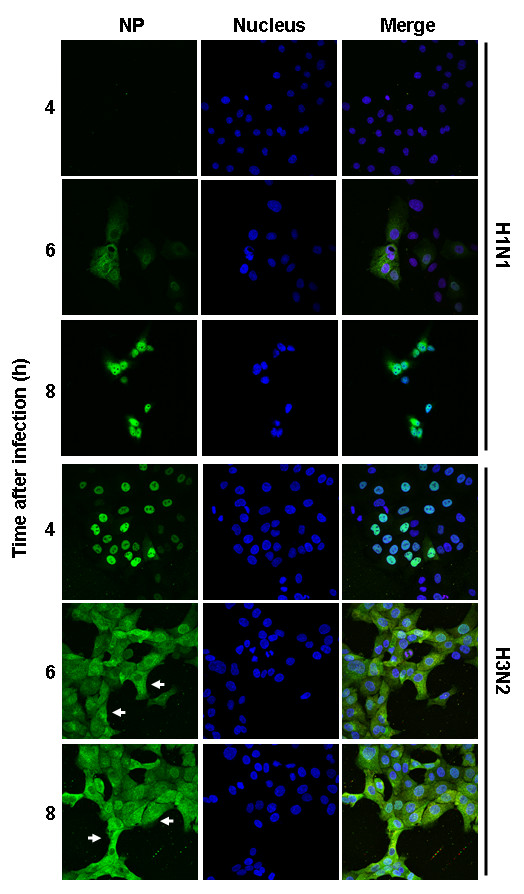
**Higher virus-induced ERK activation leads to enhanced nuclear RNP export**. MDCK cells were infected with H1N1 virus or H3N2 virus at m.o.i. = 1. RNPs were stained with anti-NP mAb and Alexa488-coupled goat anti-mouse Abs (green). The nucleus was counterstained with TO-PRO-3 (blue). Intracellular RNP localization was analyzed at indicated time points p.i. by multiphoton laser scanning microscopy. The merger of both channels is shown.

To investigate the Raf/MEK/ERK signaling-dependent nuclear RNP export, we analyzed intracellular RNP localization in cells infected with either virus. In accordance with flow cytometry analysis showing a very low amount of viral NP produced by H1N1 virus at 4 h p.i., no H1N1-NP was detected at this time point by confocal laser scanning microscopy. RNPs were localized in the cytoplasm in nearly all H3N2-infected cells at 6 and 8 h p.i., whereas in H1N1-infected cells they were localized predominantly in the nucleus or at the nuclear membrane at those time points (Fig. 3). Consequently, the H3N2 virus titers were approximately 90% higher than that of H1N1 (Fig. 2B). These results suggest an association between efficient replication and higher levels of ERK activation. The less induction of ERK activation by the H1N1 virus likely contributed to the inefficient nuclear RNP export and lower virus titers.

### Replication and growth of both influenza strains depends on their ability to activate Raf/MEK/ERK signaling

**Figure 4:**
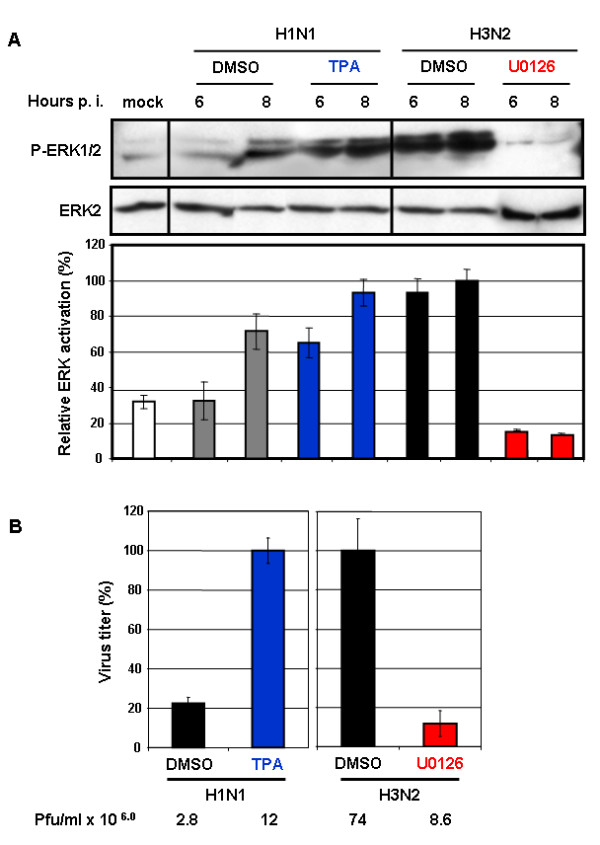
**Replication of A/HK/218847/06 (H1N1) and A/HK/218449/06 (H3N2) influenza viruses depends on ERK activation**. (A) MDCK cells were infected at m.o.i. = 1 either with H1N1 ± TPA or with H3N2 ± U0126. After Western blot analysis, ERK activation was analyzed with a mAb specific for phosphorylated ERK (P-ERK). Subsequently, loading was controlled with a mAb against ERK2. Respective bands of three independent experiments were quantified, and relative ERK activation was calculated and normalized to the loading control (mock-infected, white bar). Virus types as well as the time of analysis post-infection (p.i.) are indicated. (B) MDCK cells were infected at m.o.i. = 1 either with H1N1 ± TPA or with H3N2 ± U0126, and the supernatant was harvested 9 h later. The mean virus titers are given in percent as well as plaque forming units/ml. The error bars were derived from three independent experiments.

**Figure 5:**
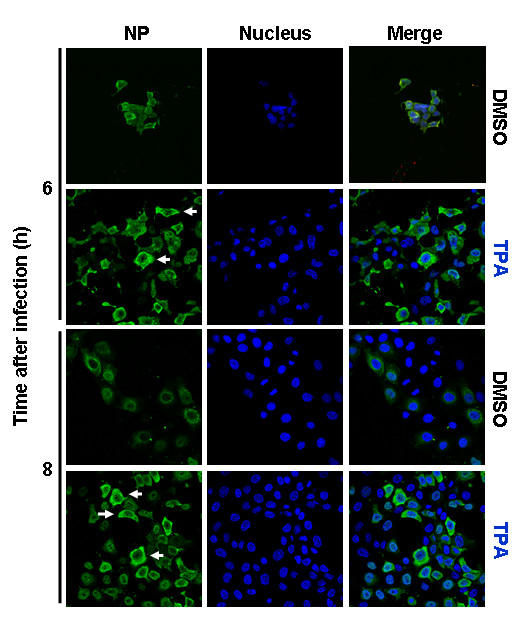
**Stimulation of MAPK pathway enhances nuclear RNP export of A/HK/218847/06 (H1N1) influenza virus**. MDCK cells were infected with H1N1 ± TPA at m.o.i. = 1. RNPs were stained with anti-NP mAb and Alexa488-coupled goat anti-mouse Abs (green). The nucleus was counterstained with TO-PRO-3 (blue). Intracellular RNP localization was analyzed at indicated time points p.i. by multiphoton laser scanning microscopy. The merger of both channels is shown.

The Raf/MEK/ERK signal cascade can be activated by either protein kinase C alpha (PKCα)-dependent or Ras-dependent pathways [24]. Upon their activation, both signal transmitters mediate phosphorylation of the kinase Raf, which further activates ERK via MEK. Thereafter, phosphorylated ERK translocates to the nucleus to phosphorylate a variety of substrates [11,12,14]. To verify if the observed difference in ERK activation between H3N2 and H1N1 viruses indeed involved MAPK signaling, we artificially enhanced or reduced the activation of MAPK signaling by applying TPA, which is a strong PKC activator and the specific MEK inhibitor U0126, respectively. In H1N1-infected cells (m.o.i. = 1), TPA markedly enhanced ERK activation at 6 h and 8 h p.i. (Fig. 4A), as well as cytoplasmic RNP localization at both time points (Fig. 5). Consequently, the virus titers increased nearly 80% (Fig. 4B). Because very little viral NP was synthesized during the first 4 h of H1N1 infection, no effect of TPA on nuclear RNP export could be seen during that time.

**Figure 6:**
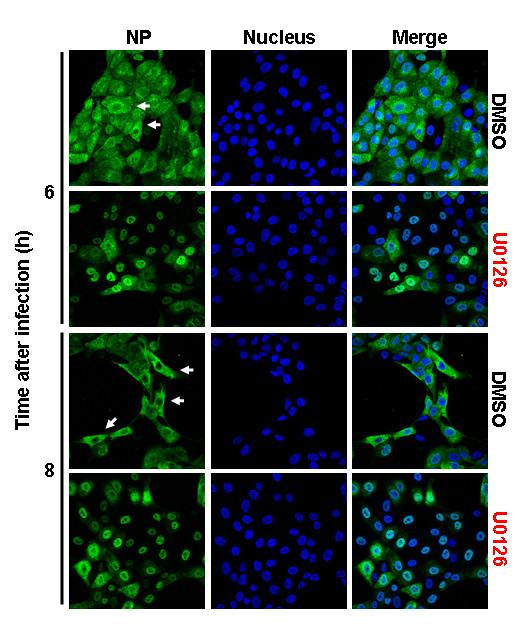
**Inhibition of MAPK pathway retards nuclear RNP export of A/HK/218449/06 (H3N2) influenza virus**. MDCK cells were infected with H3N2 ± U0126 at m.o.i. = 1. RNPs were stained with anti-NP mAb and Alexa488-coupled goat anti-mouse Abs (green). The nucleus was counterstained with TO-PRO-3 (blue). Intracellular RNP localization was analyzed at indicated time points p.i. by multiphoton laser scanning microscopy. The merger of both channels is shown.

We also assessed the effect of blocking ERK activity on H3N2-infected cells. The levels of ERK phosphorylation in H3N2-infected cells dramatically decreased (Fig. 4A). As a result, the nucleocytoplasmic transport of viral RNPs out of the nucleus during late infection was strongly suppressed (Fig. 6) and virus titers were reduced by approximately 90% (Fig. 4B). These results further support that the difference in the replication efficiency of the H1N1 and H3N2 viruses used in this study is caused on their ability to induce ERK activation.

### H3N2 influenza virus expresses more HA protein, which accumulates on the cell surface

**Figure 7:**
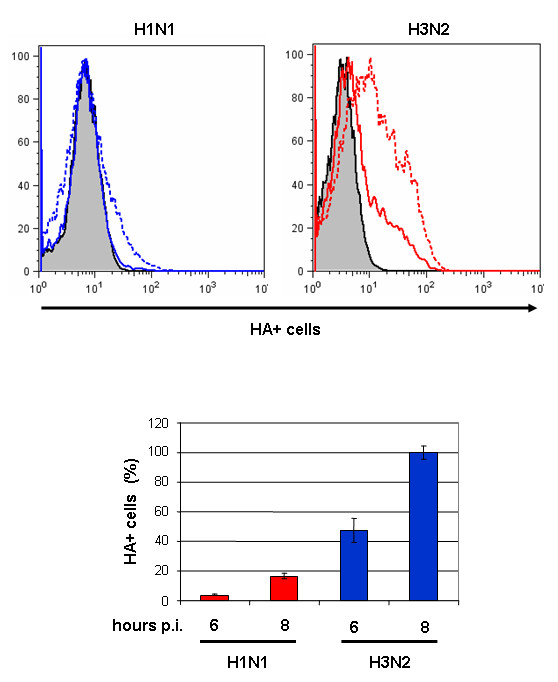
**HA surface expression of A/HK/218847/06 (H1N1) and A/HK/218449/06 (H3N2) influenza viruses**. MDCK cells were infected with either virus at m.o.i. = 1. The percentages of HA^+ ^cells were measured by FACS using a specific anti-HA mAb. In the histograms, the gray area represents the negative control; the percentage of HA^+ ^cells at 6 h p.i. (solid lines) and 8 h p.i. (dashed lines) are indicated. The bar graph shows the mean data from three independent experiments.

**Figure 8:**
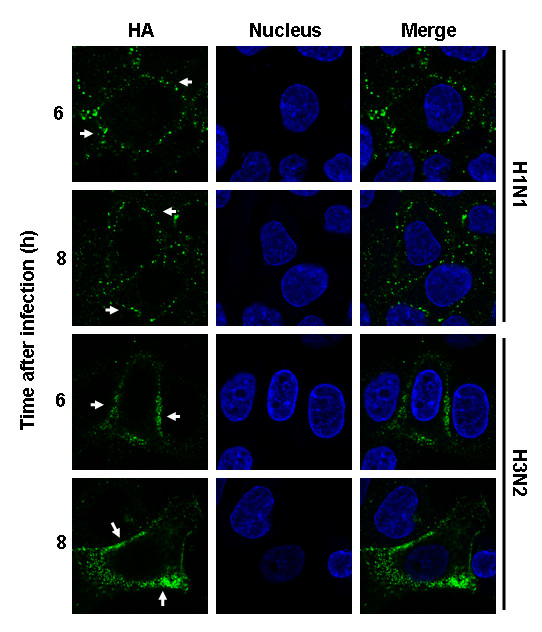
**HA surface expression of A/HK/218847/06 (H1N1) and A/HK/218449/06 (H3N2) influenza viruses**. MDCK cells were infected with either virus at m.o.i. = 1. HAs were stained with anti-HA mAb and Alexa488-coupled goat anti-mouse Abs (green). The nuclei were counterstained with TO-PRO-3 (blue). The HA surface expression was analyzed at indicated time points by confocal laser scanning microscopy. The merger of both channels is shown.

We recently showed that membrane accumulation of the HA protein triggers the activation of MAPK signaling [24]. In this study, we therefore analyzed the expression of HA on the surface of MDCK cells infected with either virus (m.o.i. = 1). The HA surface expression was measured at different time points late during virus replication. To ensure that the anti-HA antibody bound only to the HA protein on the cell surface and not to cytoplasmic HA, cells were fixed but not permeabilized. Flow cytometry (FACS) analysis showed a substantial difference in the amount of HA that accumulated on the cell membranes at 6 h and 8 h p.i.. 40% and 80% more membrane exposed HA was found on H3N2-infected cells at 6 h and 8 h p.i., respectively (*P *= 6.48 × 10^-4 ^and 5.23 × 10^-6^) (Fig. 7). To prove that these measures were indeed HA at the cell membrane and not cytoplasmic staining, we performed IFAs. The IFA data indicated that the HA proteins of both viruses were transported to the cell membrane, and in accordance with the data from the FACS analysis, the H3N2-infected cells showed more HA protein localized on the cell membrane (Fig. 8) than did the H1N1-infected cells. IFA analysis at 6 h and 8 h p.i. showed that the level of HA expression on the surface of H3N2-infected cells increased, whereas that of H1N1-infected cells was constant. These data clearly demonstrate that a greater amount of the H3N2-HA accumulates on the cell membrane compared with that of the H1N1-HA and suggest that the amount of the H3N2-HA perpetually increases during viral infection.

### Viral polymerase genes PB1 and PB2 of A/HK/218449/06 (H3N2) influenza virus exhibit higher polymerase activity than their counterparts in the H1N1 virus

The H3N2 virus replicated more efficiently in MDCK cells than did the H1N1 strain, and viral polymerase genes have been shown to contribute to virus growth and infectivity [25-28]. Therefore, we analyzed the potential role of these genes and the proteins they encode in more detail. To investigate whether the H3N2 viral polymerase genes possess higher activity than those of the H1N1 subtype, we performed a luciferase assay using a minigenome system. The pol I-driven plasmid encoding the luciferase gene was cotransfected into the human embryonic kidney cell line 293T HEK with pol I/pol II-responsive plasmids that express the viral PB1, PB2, PA, and NP proteins of the H1N1 or H3N2 virus. After 24 h, luciferase activity was assayed in cell extracts.

**Figure 9:**
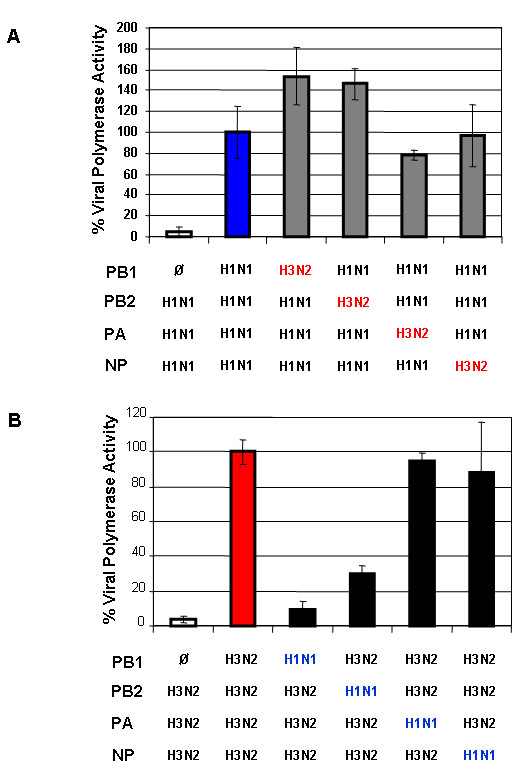
**Viral polymerase activity of A/HK/218847/06 (H1N1) and A/HK/218449/06 (H3N2) influenza viruses**. Polymerase activity was assayed in a minigenome system using a viral UTR-driven luciferase reporter gene. The 293T cells were transfected with plasmids containing PB2, PB1, PA, and NP genes from either H1N1 (A) or H3N2 (B) plus a luciferase reporter plasmid. Plasmids that did not contain PB1 were used as negative controls (white bars). After 24 h, luciferase activity was assayed in cell extracts. Results presented represent the means ± SE from three independent transfections.

To test which viral protein in the RNP complexes affect viral polymerase activity the most, we exchanged each plasmid encoding PB1, PB2, PA, or NP of both viruses. Transfection without the PB1 plasmid was also assayed as an indication for background level of non-specific luciferase expression. The relative polymerase activity of the wild type H3N2 was higher than that of the wild type H1N1. The values obtained from the transfections comprising the wild type system of each virus are individually set as 100%. Replacing H1N1 PB1 or PB2 with those genes from the H3N2 virus significantly increased the viral polymerase activity of the H1N1 virus by about 35% (Fig. 9A). Conversely, substitution of H3N2-PB1 or PB2 with those genes from the H1N1 virus reduced the polymerase activity by 91% and 70%, respectively (Fig. 9B). Replacement of the polymerase genes PA and NP did not affect the viral polymerase activity of either virus. These results demonstrated that polymerase complex of H3N2 and H1N1 differed substantially in their replication/transcription activity and that the H3N2-PB1 and PB2 contributes to higher viral polymerase activity observed between these two viruses.

### PB1 protein of A/HK/218449/06 (H3N2) influenza virus induces greater levels of ERK phosphorylation, which enhances cytoplasmic localization of the RNP complexes

**Figure 10:**
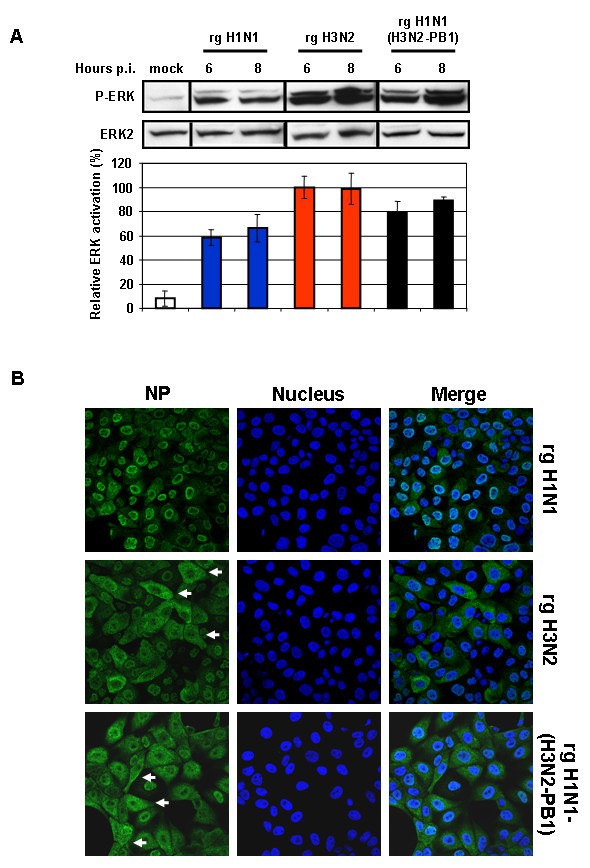
**The PB1 gene of the A/HK/218449/06 (H3N2) influenza virus contributes to ERK activation and enhanced nuclear RNP export**. (A) MDCK cells were infected at m.o.i. = 1 with reverse genetics (rg)-rescued rgH1N1, rgH3N2, or rgH1N1(H3N2-PB1). After Western blot analysis, ERK activation was analyzed with a P-ERK-specific mAb. Subsequently, loading was controlled with an anti-ERK2 mAb. Respective bands of three independent experiments were quantified, and relative ERK activation was calculated and normalized to the loading control (mock, white bar). Virus types and the time of analysis post infection (p.i.) are indicated. (B) MDCK cells were infected at m.o.i. = 1 with rgH1N1, rgH3N2, or rgH1N1(H3N2-PB1). RNPs were stained with anti-NP mAb and Alexa488-coupled goat anti-mouse Abs (green). The nucleus was counterstained with TO-PRO-3 (blue). Intracellular RNP localization was analyzed by confocal laser scanning microscopy at 6 h p.i. The merger of both channels is shown.

**Figure 11:**
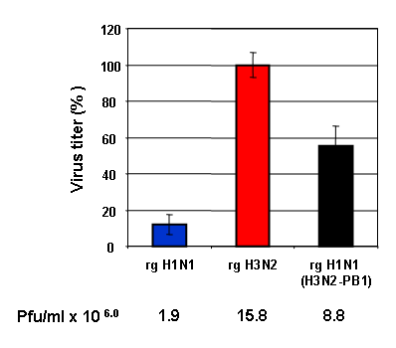
**Virus titers of recombinant influenza viruses**. MDCK cells were infected with rgH1N1, rgH3N2, or rgH1N1(H3N2-PB1) at m.o.i. = 1, and the supernatant was harvested at 9 h p.i. The mean virus titers are given in percent as well as plaque forming units/ml. The error bars were derived from three independent experiments.

The PB1 and PB2 genes appeared to have the most influence on viral polymerase activity. Since PB1 plays a central role in the catalytic activities of the RNA-dependent RNA polymerases [29], we focused on the PB1 gene to further investigate whether differences in the viral polymerase activity of H1N1 and H3N2 viruses correlate with their ability to activate the Raf/MEK/ERK signaling. To this point we used the eight-plasmid reverse genetics system [30] to generate recombinant influenza viruses to assess the potential role of the PB1 protein in virus-induced ERK activation. Recombinant viruses rgH1N1, rgH3N2 and rgH1N1/H3N2-PB1 were generated. The recombinant virus with H3N2 background possessing the H1N1-PB1 gene (rgH3N2/H1N1-PB1) could not be rescued, which might be due to gene incompatibility resulting in low rescue efficiency under these experimental conditions. The rescued H1N1 virus possessing the H3N2-PB1 (rgH1N1/H3N2-PB1) induced greater ERK phosphorylation (Fig. 10A) resulting in increased nuclear RNP export (Fig. 10B) and higher virus titers compared with that caused by rgH1N1 virus (Fig. 11). Only low levels of phosphorylated ERK were detectable in the rgH1N1-infected cells at 6 h p.i., whereas infection with rgH3N2 or rgH1N1/H3N2-PB1 significantly upregulated the virus-induced ERK activation at 6 h p.i. (Fig. 10A). Analysis of intracellular RNP localization showed that substantial export of nuclear RNP had already occurred at 6 h p.i. in cells infected with rgH3N2 or rgH1N1/H3N2-PB1, whereas the majority of the RNP complexes of rgH1N1-infected cells remained in the nucleus or at the nuclear membrane at that time point (Fig. 10B). Even though the virus titers of rgH1N1/H3N2-PB1 was lower than that of rgH3N2 at 6 h p.i., it was substantially higher than that of rgH1N1. These data demonstrate that the H3N2-PB1 protein contributes to the activation of the Raf/MEK/ERK signal cascade.

## Discussion

We compared the viral replication efficiency of two strains of IVAs isolated from two different patients in Hong Kong in 2006. The isolated H3N2 subtype replicated more efficiently than the H1N1 in MDCK cells. Interestingly, growth capacity was related to the IVA's ability to activate the Raf/MEK/ERK (MAPK) signal cascade. The H3N2 virus upregulated MAPK signaling better than did the H1N1 virus. Accordingly, stimulation of MAPK signaling with TPA, a strong kinase activator, increased the H1N1 virus titers. In contrast, treatment of H3N2-infected cells with the specific MEK inhibitor U0126 abolished ERK activation and severely reduced the virus titers. These data show that replication of both viruses strongly depends on their ability to activate the MAPK signaling. Cell treatment with TPA or U0126 did not affect the synthesis of viral NPs at 6 and 8 h p.i. (data not shown). This finding showed that changes in virus titers, at least in part, are indeed influenced by nuclear export efficiency of the RNPs.

Moreover, many studies have shown that the polymerase genes of more replication-efficient influenza viruses play a central role in virulence and virus replication [25,31,27]. The H3N2-PB1 and PB2 significantly contributed to higher polymerase activity. We further studied the importance of the viral PB1 polymerase for virus-induced ERK activation, because (i) replacing the PB1 protein of each virus most significantly increased or decreased the polymerase activity and (ii) the PB1 subunit plays a central role in the catalytic activities of the RNA polymerase as it contains the conserved motifs characteristic of RNA-dependent RNA polymerases and is directly involved in RNA chain elongation [29]. For this purpose, recombinant influenza viruses (rgH1N1, rgH3N2 and rgH1N1/H3N2-PB1) were generated to assess the role of PB1. Our data showed rgH1N1/H3N2-PB1 virus elevated ERK phosphorylation, thereby causing enhanced export of nuclear RNPs and increased virus titers as compared to that of the rgH1N1 virus. However, the ERK activation induced by rgH1N1/H3N2-PB1 is still weaker than that induced by rgH3N2. Therefore, although the H3N2-PB1 protein appears to contribute to elevated ERK activation, other viral proteins (e.g., HA) from wild-type H3N2 may still be required for optimal ERK activation. On the other hand, PB2 and particularly PB1 of H1N1 dramatically reduced the transcription/replication activity of H3N2. This may explain why no recombinant virus with an H3N2 background possessing H1N1-PB1 could be rescued. In contrast, replacement of the H1N1-PB1 with that of H3N2 increased the viral polymerase activity. These findings demonstrate for the first time the relation between viral polymerase activity and activation of MAPK signaling. In addition to the crucial function of PB1, the PB2 subunit is responsible for recognition and binding of the cap structure of host mRNAs [32,33]. The role of the PA subunit in the transcription and replication of vRNA is less well established. However, it has been shown that the PA subunit is required for efficient nuclear accumulation of the PB1 protein [34]. Based on our data and this observation, it would also be interesting to further study the possible contribution of PB2 in virus-induced MAPK activation.

**Figure 12:**
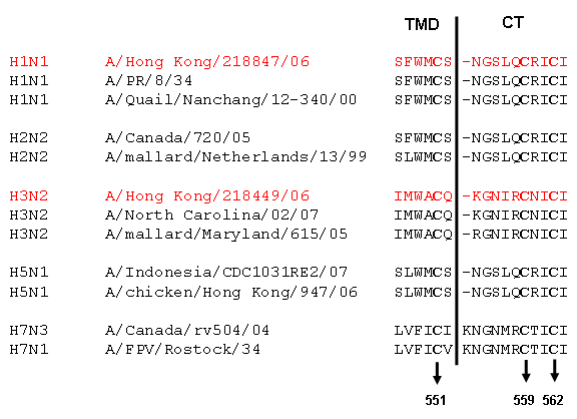
**Comparison of the carboxy-terminal amino acid sequences of different HA subtypes**. Dashes are inserted to give maximum sequence homology. The conserved cysteine residues serving as acylation (palmitoylation) sites are shown in bold face. The HA sequences of A/HK/218847/06 (H1N1) and A/HK/218449/06 (H3N2) are highlighted in red. TMD, transmembrane domain; CT, cytoplasmic tail

Previously, we showed that a tight association of viral HA with lipid raft domains localized in the cell membrane is crucial for activating the virus-induced MAPK signal cascade [24]. Three highly conserved cysteine residues in the HA cytoplasmic tail of A/FPV/Rostock/34 (H7N1) at positions 551, 559, and 562 serve as acylation (palmitoylation) sites that are important for HA/lipid raft association [35], ERK activation, nuclear RNP export, and subsequently infectivity [24]. Insufficient transport of HA from the cytoplasm to the cell surface was shown to be responsible for the low activation of ERK [24]. Like the H7N1-HA, the HAs from the two IVAs examined in this study also possess cysteine residues at these positions (Fig. 12). On the basis of this observation, we assume that the HAs of the H1N1 and H3N2 viruses used in this study should therefore be able to interact with lipid raft domains to activate the MAPK signal cascade. Unlike the H3N2 subtype, the H1N1 showed a severely reduced ability to activate ERK to the level required for efficient virus replication. FACS and IFA analyses revealed that more H3N2-HA was expressed and accumulated on the membranes of infected cells than was H1N1-HA. This finding further supports previously published data and suggests that the difference in membrane accumulation of the H3N2-HA compared to the H1N1-HA triggers higher activation of the MAPK cascade and more efficient nuclear RNP export.

Next, we tried to figure out the fundamental reasons why the H3N2 strain replicates more efficiently than the H1N1 subtype does. It is noteworthy that most of the currently circulating H5N1 strains with pandemic potential replicate very fast and exhibit high lethality in various hosts. The viral polymerase genes, particularly PB1 and PB2, contribute to the virulence of the human A/Vietnam/1203/04 (H5N1) influenza virus in mice and ferrets [27]. Sequence analysis of the two IVAs examined in the current study revealed differences in 42 amino acid (aa) residues in the PB1 genes. Interestingly, compared with the sequence of the PB1 of A/Vietnam/1203/04, that of H3N2-PB1 differs by only 21 residues, while that of the H1N1-PB1 differs by 34. Furthermore, accumulating evidence indicates that from 1918 to 1947, the human H1N1 viruses contained PB1 genes with a full-length PB1-F2, whereas beginning in 1956, human H1N1 strains contain a PB1-F2 that is truncated after codon 57 [36]. Most of the recent human H3N2 virus isolates encode an intact PB1-F2 [36]. PB1-F2 protein is encoded in the +1 open-reading frame of segment-2 RNA [10]. The C-terminal domain of PB1-F2 contains the mitochondrial signal and can trigger apoptosis in specific immune-related cells [36,37]. Zamarin et al. have demonstrated that full-length PB1-F2 contributes to the virus' pathogenesis in mice [38]. Interestingly, the PB1-F2 gene of the H3N2 virus used in this study consists of 90 aa residues (full length), whereas that of the H1N1 consists of only 57 aa. The facts that H3N2-PB1 has higher homology with H5N1-PB1 and that the PB1-F2 protein of H3N2 has a full-length sequence, may explain why the H3N2 subtype replicates more efficiently than does the H1N1 virus and induces higher activation levels of the MAPK signal cascade.

All together, our findings led us to conclude that the viral polymerase complex contributes to the activation of HA-induced MAPK signaling. Influenza virus takes advantage of this event, in turn, to optimize viral growth. Our current data suggest that higher viral polymerase activity enhances the replication and transcription of viral RNA, which leads to greater expression of the viral HA protein and its accumulation on the cell surface late during virus replication. This in turn results in stronger ERK activation and thereby to more efficient nuclear RNP export and formation of infectious progeny virions. Understanding such a mechanism essential for influenza virus replication may also be a basis for the development of therapeutic implications, such as antiviral drug that reduces the polymerase activity leading to decreased HA-membrane accumulation and declined activation of the MAPK pathway.

## Conclusion

These results showed that HK/218449/06 (H3N2) influenza virus replicates more efficiently than HK/218847/06 (H1N1) subtype does. Infection with the H3N2 strain induced higher activation levels of the Raf/MEK/ERK (MAPK) signal cascade essential for virus replication. The previous study demonstrated the role of HA as an inducer of MAPK signaling causing enhanced nuclear RNP export at late time point of infectious cycle. Applying reverse genetic systems, we could show that the viral polymerase proteins (particularly PB1 and PB2) of the H3N2 influenza virus possess higher polymerase activity and that the PB1 protein of the H3N2 influenza virus contributes to the elevated HA-induced ERK activation, increased cytoplasmic RNP localization and higher virus titers.

## Materials and methods

### Cells, viruses, and infection

Human embryonic kidney cells (293T cells) were maintained in Dulbecco's modified Eagle's medium (DMEM) supplemented with 10% fetal calf serum (FCS) and antibiotics. Madin-Darby canine kidney (MDCK) cells were kept in minimal essential medium supplemented with 10% FCS and antibiotics. All cells were cultivated at 37°C with 5% CO_2_. Human influenza viruses A/Hong Kong/218847/06 (H1N1) and A/Hong Kong/218849/06 (H3N2) were kindly provided by Dr. Malik Peiris (University of Hong Kong). We rescued the following viruses by reverse genetics (rg): rgH1N1, rgH3N2, and rgH1N1/H3N2-PB1. These five viruses were used to infect MDCK cells. Cells were washed with phosphate-buffered saline (PBS), infected at the indicated multiplicity of infection (m.o.i.), and further incubated as described previously [23,24].

### Generation of recombinant viruses by a reverse genetics system

H1N1 and H3N2 IVAs were propagated in MDCK cells. RT-PCR using gene specific primers [39] was done to amplify all eight viral genes, and viral cDNAs were inserted into dual-promoter plasmid pHW2000 [30]. All plasmids were sequenced, and QuikChange Site-Directed Mutagenesis kits (Stratagene) were used to adapt the coding sequences of the cloned fragments to the sequence identified by PCR fragment sequencing. Recombinant viruses were generated by DNA transfection of MDCK/293T cells as described [30]. The supernatant of transfected cells was used for reinfection of MDCK cells, and virus stock was prepared, sequenced, and titrated.

### Sequence analysis

Viral RNA was isolated directly from virus-containing supernatant by using an RNA isolation kit (RNeasy; QIAGEN). The universal primer set for influenza A virus was used for RT-PCR [39]. The Hartwell Center for Bioinformatics & Biotechnology at St. Jude Children's Research Hospital determined the sequence of the DNA template by using Big Dye Terminator (v.3) chemistry and synthetic oligonucleotides. Samples were analyzed on 3700 DNA analyzers (Applied Biosystems).

### Plaque assay and TCID_50_

Confluent monolayers of MDCK cells in 35-mm dishes were inoculated with 10-fold dilutions of influenza virus (in DMEM with 3% BSA and antibiotics) and incubated at 37°C for 1 h. The inoculum was removed, and cells were washed with PBS and overlaid with MEM containing 1% agarose and 0.2% serum albumin. After 3 d at 37°C, cells were stained with 0.1% crystal violet in 10% formaldehyde solution, and plaque morphology was evaluated. Plaque size was measured using fine-scale magnifying comparator (6×). To determine the 50% tissue culture infecting dose (TCID_50_), we inoculated confluent monolayers of MDCK cells in a 96-well plate with 10-fold dilutions of influenza virus and incubated them at 37°C for 1 h. After inoculum removal, cells were washed with PBS and incubated for 72 h. A 50-μl sample of supernatant was drawn from each well, transferred to a new 96-well plate, and virus was titrated by HA test with a 0.5% suspension of chicken red blood cells. The TCID_50 _was calculated by the method of Reed and Muench [40].

### Activation and inhibition of the Raf/MEK/ERK signal cascade

Activation of the Raf/MEK/ERK signal cascade was achieved by artificial stimulation of MDCK cells with 100 ng/ml 12-*O*-tetradecanoyl-phorbol-13-acetate (TPA) (Sigma) at 4 h p.i.. U0126 (50 mM), a specific MEK inhibitor (Promega), was used to inhibit ERK activity as described previously [23].

### Detection of ERK phosphorylation by Western blotting

Cell lysate was cleared by centrifugation, and protein concentration was determined by Bradford assay before the protein was subjected to SDS-PAGE. Phosphorylated ERK (P-ERK) was detected with a specific monoclonal antibody (Santa Cruz Biotechnology). After stripping bound antibodies, we detected the total ERK2 using mAbs (Santa Cruz Biotechnology). Proteins recognized by mAbs were further analyzed with peroxidase-coupled, species-specific secondary antibodies and a standard enhanced chemiluminescence reaction (Amersham Biosciences). Quantification of specific bands was done with the PC-BAS software package (Fuji).

### Confocal Laser Scanning Microscopy and Immunofluorescence Assay (IFA)

MDCK cells grown on glass coverslips were infected and incubated as indicated below. The cells were washed with PBS at the indicated time points p.i. and fixed with 4% paraformaldehyde (PFA) in PBS at room temperature (rt) for 30 min or at 4°C over night. Cells were permeabilized with 1% Triton X-100 (in PBS) at rt for 10 min. Then cells were incubated with a combination of the mouse anti-IVA NP mAb, clone AA5H (1:100) (Abcam) in PBS/3% bovine serum albumin (BSA) at rt for 1 h. The AlexaFluor488-coupled goat anti-mouse antibody (Invitrogen) was used as the secondary antibody. Cells were washed with PBS followed by double-distilled water and mounted with P-phenyldiamine (PPD) (Sigma) containing 500 nM TO-PRO-3 (Molecular Probes) for nuclear staining. Fluorescence was visualized with a multiphoton laser scanning microscope (Zeiss LSM 510 META). To analyze the expression of HA on the cell surface, cells were not permeabilized. The HA protein in infected cells was detected by anti-H1HA mAb (abcam, clone C102) or by anti-H3HA (against HA of A/Mem/1/94) mAb (St. Jude Children's Research Hospital, clone H3/94/49) and AlexaFluor488-coupled goat anti-mouse antibody as secondary antibody.

### Flow cytometry (FACS) analysis

MDCK cells were infected with either HK/218847 (H1N1) or HK/218449 (H3N2) as indicated below. Cells were incubated for 4, 6, or 8 h. Then the cells were detached with trypsin, fixed in PBS/4% PFA, permeabilized with 1% Triton X-100, and stepwise incubated with FITC-conjugated mouse anti-NP mAb, (clone IA52, 1:500; Argene INC) in PBS/3% BSA for 30 min on ice. Finally, the percentage of NP-expressing cells was determined by flow cytometry analysis using FACSCalibur (BD Biosciences). To analyze expression of HA on the cell surface, cells were not permeabilized. The HA protein in infected cells was detected by anti-H1HA mAb (abcam, clone C102) or by anti-H3HA mAb (St. Jude Children's Hospital, clone H3/94/49) and AlexaFluor488-coupled goat anti-mouse antibody as secondary antibody.

### Luciferase assays

Subconfluent monolayers of 293T cells (7.5 × 10^5 ^cells in 35-mm dishes) were transfected (Mirus Bio) with 2 μg luciferase reporter plasmid (EGFP open-reading frame in pHW72-EGFP substituted with luciferase gene [30] and a mix of PB2 (1 μg), PB1 (1 μg), PA (1 μg), and NP (2 μg) plasmids of A/HK/218847/06 (H1N1) and A/HK/218449/06 (H3N2) viruses. After 24 h, cell extracts were prepared in 500 μl lysis buffer, and luciferase levels were assayed with a Luciferase Assay System (Promega) and BD Monolight 3010 luminometer (BD Biosciences). Experiments were performed in triplicate.

## Competing interests

The author(s) declare that they have no competing interests.

## Authors' contributions

HM performed most of the experiments and wrote the manuscript. HY and JF helped to generate the expression plasmids and designed the studies. HY, RW, SP and EH contributed to scientific ideas and analysis of the data.

## References

[B1] Alexander DJ, Brown IH (2000). Recent zoonoses caused by influenza A viruses. Rev Sci Tech.

[B2] Laver WG, Air GM, Webster RG, Smith-Gill SJ (1990). Epitopes on protein antigens: misconceptions and realities. Cell.

[B3] Webster RG, Wright SM, Castrucci MR, Bean WJ, Kawaoka Y (1993). Influenza – a model of an emerging virus disease. Intervirology.

[B4] Taubenberger JK, Morens DM (2006). Influenza revisited. Emerg Infect Dis.

[B5] Taubenberger JK, Morens DM (2006). 1918 Influenza: the mother of all pandemics. Emerg Infect Dis.

[B6] Kaji M, Watanabe A, Aizawa H (2003). Differences in clinical features between influenza A H1N1, A H3N2, and B in adult patients. Respirology.

[B7] Krug RMA-K, F V, Julkunen I, Katze MG (1989). Expression and replication of the influenza virus genome.

[B8] Lamb RA (1989). The influenza viruses.

[B9] Simons K, Toomre D (2000). Lipid rafts and signal transduction. Nat Rev Mol Cell Biol.

[B10] Chen W, Calvo PA, Malide D, Gibbs J, Schubert U, Bacik I, Basta S, O'Neill R, Schickli J, Palese P (2001). A novel influenza A virus mitochondrial protein that induces cell death. Nat Med.

[B11] Chen Z, Gibson TB, Robinson F, Silvestro L, Pearson G, Xu B, Wright A, Vanderbilt C, Cobb MH (2001). MAP kinases. Chem Rev.

[B12] Peyssonnaux C, Eychene A (2001). The Raf/MEK/ERK pathway: new concepts of activation. Biol Cell.

[B13] Zugasti O, Rul W, Roux P, Peyssonnaux C, Eychene A, Franke TF, Fort P, Hibner U (2001). Raf-MEK-Erk cascade in anoikis is controlled by Rac1 and Cdc42 via Akt. Mol Cell Biol.

[B14] Pearson G, Robinson F, Beers Gibson T, Xu BE, Karandikar M, Berman K, Cobb MH (2001). Mitogen-activated protein (MAP) kinase pathways: regulation and physiological functions. Endocr Rev.

[B15] Duncia JV, Santella JB, Higley CA, Pitts WJ, Wityak J, Frietze WE, Rankin FW, Sun JH, Earl RA, Tabaka AC (0126). MEK inhibitors: the chemistry and biological activity of U its analogs, and cyclization products. Bioorg Med Chem Lett.

[B16] Ehrhardt C, Kardinal C, Wurzer WJ, Wolff T, von Eichel-Streiber C, Pleschka S, Planz O, Ludwig S (2004). Rac1 and PAK1 are upstream of IKK-epsilon and TBK-1 in the viral activation of interferon regulatory factor-3. FEBS Lett.

[B17] Planz O, Pleschka S, Ludwig S (2001). MEK-specific inhibitor U0126 blocks spread of Borna disease virus in cultured cells. J Virol.

[B18] Popik W, Pitha PM (2000). Exploitation of cellular signaling by HIV-1: unwelcome guests with master keys that signal their entry. Virology.

[B19] Ludwig S, Pleschka S, Planz O, Wolff T (2006). Ringing the alarm bells: signalling and apoptosis in influenza virus infected cells. Cell Microbiol.

[B20] Ludwig S, Wolff T, Ehrhardt C, Wurzer WJ, Reinhardt J, Planz O, Pleschka S (2004). MEK inhibition impairs influenza B virus propagation without emergence of resistant variants. FEBS Lett.

[B21] Olschlager V, Pleschka S, Fischer T, Rziha HJ, Wurzer W, Stitz L, Rapp UR, Ludwig S, Planz O (2004). Lung-specific expression of active Raf kinase results in increased mortality of influenza A virus-infected mice. Oncogene.

[B22] Wurzer WJ, Ehrhardt C, Pleschka S, Berberich-Siebelt F, Wolff T, Walczak H, Planz O, Ludwig S (2004). NF-kappaB-dependent induction of tumor necrosis factor-related apoptosis-inducing ligand (TRAIL) and Fas/FasL is crucial for efficient influenza virus propagation. J Biol Chem.

[B23] Pleschka S, Wolff T, Ehrhardt C, Hobom G, Planz O, Rapp UR, Ludwig S (2001). Influenza virus propagation is impaired by inhibition of the Raf/MEK/ERK signalling cascade. Nat Cell Biol.

[B24] Marjuki H, Alam MI, Ehrhardt C, Wagner R, Planz O, Klenk HD, Ludwig S, Pleschka S (2006). Membrane accumulation of influenza A virus hemagglutinin triggers nuclear export of the viral genome via protein kinase Calpha-mediated activation of ERK signaling. J Biol Chem.

[B25] Gabriel G, Dauber B, Wolff T, Planz O, Klenk HD, Stech J (2005). The viral polymerase mediates adaptation of an avian influenza virus to a mammalian host. Proc Natl Acad Sci USA.

[B26] Hatta M, Gao P, Halfmann P, Kawaoka Y (2001). Molecular basis for high virulence of Hong Kong H5N1 influenza A viruses. Science.

[B27] Salomon R, Franks J, Govorkova EA, Ilyushina NA, Yen HL, Hulse-Post DJ, Humberd J, Trichet M, Rehg JE, Webby RJ (2006). The polymerase complex genes contribute to the high virulence of the human H5N1 influenza virus isolate A/Vietnam/1203/04. J Exp Med.

[B28] Subbarao EK, London W, Murphy BR (1993). A single amino acid in the PB2 gene of influenza A virus is a determinant of host range. J Virol.

[B29] Biswas SK, Nayak DP (1994). Mutational analysis of the conserved motifs of influenza A virus polymerase basic protein 1. J Virol.

[B30] Hoffmann E, Neumann G, Hobom G, Webster RG, Kawaoka Y (2000). "Ambisense" approach for the generation of influenza A virus: vRNA and mRNA synthesis from one template. Virology.

[B31] Li Z, Chen H, Jiao P, Deng G, Tian G, Li Y, Hoffmann E, Webster RG, Matsuoka Y, Yu K (2005). Molecular basis of replication of duck H5N1 influenza viruses in a mammalian mouse model. J Virol.

[B32] Fechter P, Mingay L, Sharps J, Chambers A, Fodor E, Brownlee GG (2003). Two aromatic residues in the PB2 subunit of influenza A RNA polymerase are crucial for cap binding. J Biol Chem.

[B33] Li ML, Rao P, Krug RM (2001). The active sites of the influenza cap-dependent endonuclease are on different polymerase subunits. Embo J.

[B34] Fodor E, Crow M, Mingay LJ, Deng T, Sharps J, Fechter P, Brownlee GG (2002). A single amino acid mutation in the PA subunit of the influenza virus RNA polymerase inhibits endonucleolytic cleavage of capped RNAs. J Virol.

[B35] Wagner R, Herwig A, Azzouz N, Klenk HD (2005). Acylation-mediated membrane anchoring of avian influenza virus hemagglutinin is essential for fusion pore formation and virus infectivity. J Virol.

[B36] Cano R, Jarraud S, Pardos J, Campese C, Pelaz C (2006). Influenza A Virus PB1-F2 Gene. Emerging Infectious Diseases.

[B37] Zell R, Krumbholz A, Wutzler P (2006). Influenza A virus PB1-F2 gene. Emerg Infect Dis.

[B38] Zamarin D, Ortigoza MB, Palese P (2006). Influenza A virus PB1-F2 protein contributes to viral pathogenesis in mice. J Virol.

[B39] Hoffmann E, Stech J, Guan Y, Webster RG, Perez DR (2001). Universal primer set for the full-length amplification of all influenza A viruses. Arch Virol.

[B40] Reed LJMH (1938). A simple method for estimating fifty percent endpoints. Am J Hyg.

